# The Genome of the Beluga Whale (*Delphinapterus leucas*)

**DOI:** 10.3390/genes8120378

**Published:** 2017-12-11

**Authors:** Steven J. M. Jones, Gregory A. Taylor, Simon Chan, René L. Warren, S. Austin Hammond, Steven Bilobram, Gideon Mordecai, Curtis A. Suttle, Kristina M. Miller, Angela Schulze, Amy M. Chan, Samantha J. Jones, Kane Tse, Irene Li, Dorothy Cheung, Karen L. Mungall, Caleb Choo, Adrian Ally, Noreen Dhalla, Angela K. Y. Tam, Armelle Troussard, Heather Kirk, Pawan Pandoh, Daniel Paulino, Robin J. N. Coope, Andrew J. Mungall, Richard Moore, Yongjun Zhao, Inanc Birol, Yussanne Ma, Marco Marra, Martin Haulena

**Affiliations:** 1 Canada’s Michael Smith Genome Sciences Centre, British Columbia Cancer Agency, Vancouver, BC V5Z 4E6, Canada; gtaylor@bcgsc.ca (G.A.T.); sichan@bcgsc.ca (S.C.); rwarren@bcgsc.ca (R.L.W.); shammond@bcgsc.ca (S.A.H.); sbilobram@bcgsc.ca (S.B.); samjones@bcgsc.ca (S.J.J.); ktse@bcgsc.ca (K.T.); ili@bcgsc.ca (I.L.); dorothyc@bcgsc.ca (D.C.); kmungall@bcgsc.ca (K.L.M.); cchoo@bcgsc.ca (C.C.); aally@bcgsc.ca (A.A.); ndhalla@bcgsc.ca (N.D.); atam@bcgsc.ca (A.K.Y.T.); armellet@bcgsc.ca (A.T.); hkirk@bcgsc.ca (H.K.); ppandoh@bcgsc.ca (P.P.); dpaulino@bcgsc.ca (D.P.); rcoope@bcgsc.ca (R.J.N.C.); amungall@bcgsc.ca (A.J.M.); rmoore@bcgsc.ca (R.M.); yzhao@bcgsc.ca (Y.Z.); ibirol@bcgsc.ca (I.B.); yma@bcgsc.ca (Y.M.); mmarra@bcgsc.ca (M.M.); 2 Department of Molecular Biology and Biochemistry, Simon Fraser University, Burnaby, BC V5A 1S6, Canada; 3 Department of Medical Genetics, University of British Columbia, Vancouver, BC V6T 1Z3, Canada; 4 Department of Earth, Ocean & Atmospheric Sciences, University of British Columbia, Vancouver, BC V6T 1Z4, Canada; gmordecai@eoas.ubc.ca (G.M.); suttle@science.ubc.ca (C.A.S.); amy.chan@ubc.ca (A.M.C.); 5 Institute for the Oceans & Fisheries, University of British Columbia, Vancouver, BC V6T 1Z4, Canada; 6 Department of Microbiology & Immunology, University of British Columbia, Vancouver, BC V6T 1Z3, Canada; 7 Department of Botany, University of British Columbia, Vancouver, BC V6T 1Z4, Canada; 8 Fisheries and Oceans Canada, Molecular Genetics Section, Pacific Biological Station, Nanaimo, BC V9R 5K6, Canada; kristi.miller@dfo-mpo.gc.ca (K.M.M.); angela.dchulze@dfo-mpo.gc.ca (A.S.); 9 Vancouver Aquarium, Vancouver, BC V6G 3E2, Canada; martin.haulena@ocean.org

**Keywords:** genome, genome assembly, beluga whale, *Delphinapterus leucas*, Cetacea

## Abstract

The beluga whale is a cetacean that inhabits arctic and subarctic regions, and is the only living member of the genus *Delphinapterus*. The genome of the beluga whale was determined using DNA sequencing approaches that employed both microfluidic partitioning library and non-partitioned library construction. The former allowed for the construction of a highly contiguous assembly with a scaffold N50 length of over 19 Mbp and total reconstruction of 2.32 Gbp. To aid our understanding of the functional elements, transcriptome data was also derived from brain, duodenum, heart, lung, spleen, and liver tissue. Assembled sequence and all of the underlying sequence data are available at the National Center for Biotechnology Information (NCBI) under the Bioproject accession number PRJNA360851A.

## 1. Introduction

The beluga or white whale has a circumpolar distribution in arctic and subarctic regions [1]. It belongs to the two member cetacean family Monodontidae, along with the narwhal (*Monodon monoceros*). The beluga whale is characteristically white and lacks a dorsal fin. The latter is presumed to be an adaptation to coping with under-ice conditions and to preserve heat [2]. Here, we present the genomic sequence and gene annotation resources for the beluga whale. This genome assembly will further aid in the comparative genomic analysis of marine mammals, complementing the cetacean genome resources of the orca (*Orinicus orca*), minke whale (*Balaenoptera acutorostrata*), and bottlenose dolphin (*Tursiops truncatus*).

## 2. Methods, Results and Discussion

**Figure 1:**
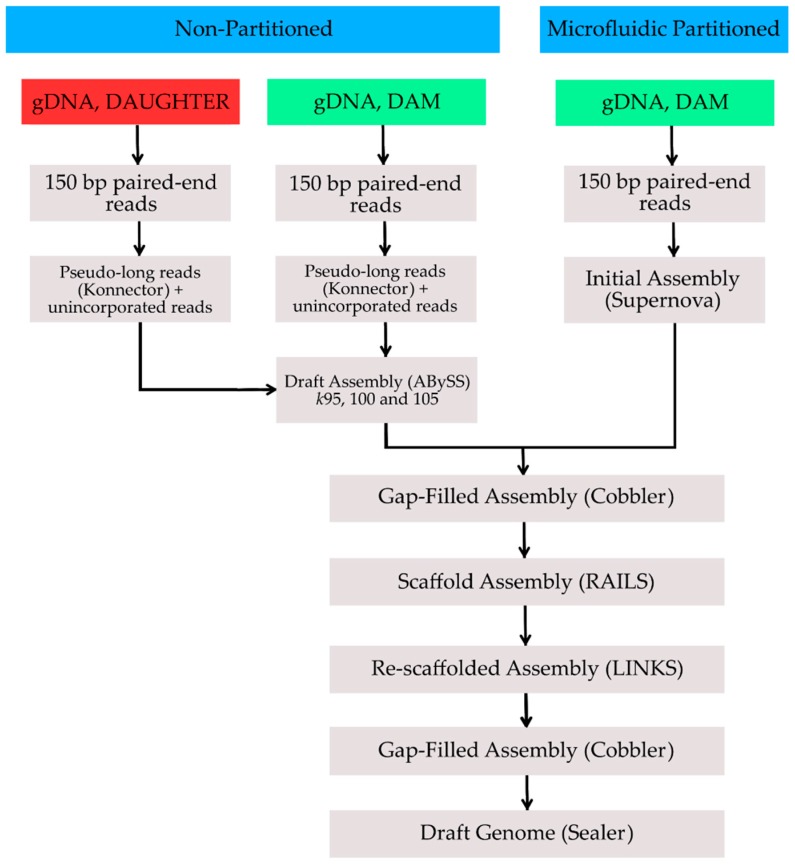
Genome assembly workflow. gDNA: Genomic DNA.

The genome was assembled using paired-end reads that were sequenced from both standard Illumina and microfluidic partitioned genomic DNA libraries (Figure 1). All genomic sequence was generated using the Illumina HiSeq X platform (Illumina, San Diego, CA, USA) at Canada’s Michael Smith Genome Sciences Centre (Vancouver, BC, Canada). DNA samples were obtained from blood derived from two related individuals (dam and adult daughter) descended originally from near Churchill, MB, Canada.

For the generation of the microfluidic partitioned library (using the Chromium System, 10x Genomics Inc., Pleasanton, CA, USA) we generated DNA of high molecular weight (predominately > 50 kbp) from individual GAN/ISIS:26980492/103006 (dam). Briefly, the genomic DNA (gDNA) was extracted using the QIAGEN MagAttract high molecular weight DNA Kit (QIAGEN, Germantown, MD, USA) using the DNA Extraction protocol from the manufacturer (Chromium Genome Reagent Kits Version 2 User Guide). The integrity of the gDNA was confirmed using pulsed-field gel electrophoresis (PFGE). Using the Chromium Controller instrument (10x Genomics) fitted with a micro-fluidic Genome Chip (PN-120216) (10x Genomics), a library of Genome Gel Beads was combined with 1 ng of gDNA, Master Mix and partitioning oil to create Gel Bead-In-EMulsions (GEMs). The GEMs were subjected to an isothermic amplification step and bar-coded DNA fragments were recovered for Illumina library construction, following the Chromium Genome Reagent Kits Version 2 User Guide (PN-120229) (10x Genomics). Quantitative polymerase chain reaction (qPCR) was performed to assess library yield and an Agilent 2100 Bioanalyzer DNA 1000 chip (Agilent Technologies, Inc., Waldbronn, Germany) was used to determine the library size range and distribution. The library was sequenced on the Illumina HiSeq X sequencer, using paired-end sequencing to produce 150 bp reads at an estimated 60-fold redundant sequence coverage.

Non-partitioned paired-end DNA sequencing libraries were produced from both individuals, dam and daughter. DNA was extracted from 200 µL peripheral blood using a QIAamp DNA Blood Mini kit (QIAGEN), automated on a QIAcube (QIAGEN) and quantified using a Quant-iT double-stranded DNA, High Sensitivity assay (Thermo Fisher Scientific, Waltham, MA, USA). Genomic DNA (500 ng) was used to construct whole genome PCR-free libraries for each whale. Briefly, DNA was sheared to 300–600 bp by acoustic sonication for 90 s (Covaris Inc., Woburn, MA, USA). Sheared DNA was end-repaired and size selected using PCRClean DX magnetic beads (Aline Biosciences, Woburn, MA, USA) targeting ≈450 bp fragments. After 3’-A-tailing, full-length Illumina TruSeq adapters (Illumina) were ligated. Libraries were purified using ALINE beads and fragment sizes were assessed using an aliquot of PCR amplified library DNA on an Agilent 2100 Bioanalyzer DNA1000 chip. PCR-free library concentrations were quantified using a qPCR Library Quantification kit (KAPA, KK4824) (Kapa Biosystems, Wilmington, MA, USA). Each genome library was sequenced on a single lane of the HiSeq X instrument with paired-end 150 bp reads, with each also generating an estimated 60-fold redundant sequence coverage.

**Figure 2:**
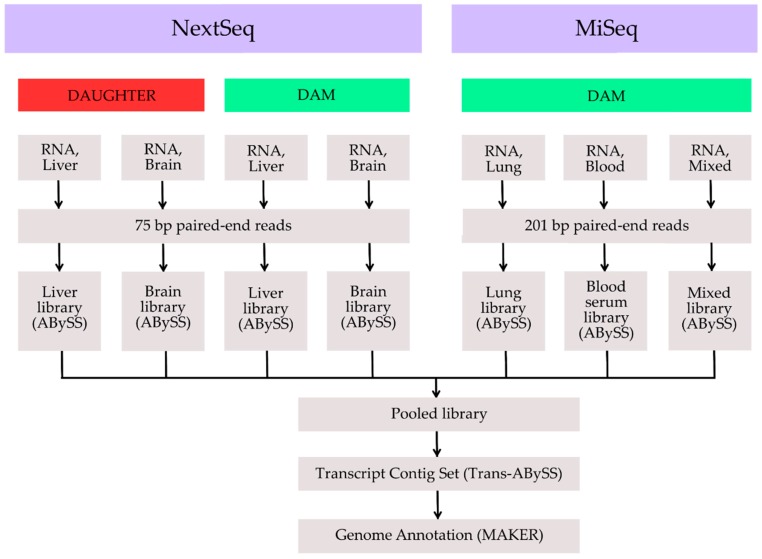
Transcriptome assembly workflow.

To aid in the subsequent annotation of the genome and provide a transcriptome resource, RNA sequencing (RNA-Seq) was performed using both the Illumina NextSeq and the Illumina MiSeq optimizing for read depth and read length for each platform, respectively (Figure 2).

For the Illumina NextSeq total nucleic acids were extracted from the liver and brain tissue from each individual using the ALINE EvoPure RNA Isolation Kit (Aline Biosciences). Following DNase I treatment, 250 ng total RNA was used for ribosomal (rRNA) removal using the NEBNext rRNA Depletion Kit (New England Biolabs, Inc., Ipswich, MA, USA). Complementary DNA (cDNA) synthesis and paired-end strand specific RNA-Seq library construction were performed according to our modified protocols, the main modification includes the random primed cDNA synthesis using Maxima H Minus 1st strand cDNA synthesis kit (Thermo Fisher) with Actinomycin D and NEBNext directional second strand cDNA module (New England Biolabs, Inc.). Libraries were pooled and sequenced, generating 75 bp paired-end reads.

RNA for the Illumina MiSeq was sequenced from blood serum, lung, and a mixture of tissues comprising of equal amounts of brain, duodenum, heart, liver, lung, and spleen. These tissues were all extracted from the dam. Homogenization using Tri-reagent was performed in a Mixer Mill (QIAGEN) on replicates of the various tissues. Total RNA extractions on 100 µL of the aqueous layers for each tissue replicate, as well as the pooled sample containing equal volumes of aqueous layers from tissues, were performed using the Magma-96 for Microarrays RNA kit (Ambion, Inc., Austin, TX, USA) spin protocol with the addition of a TURBO DNase step, on a Biomek NXP (Beckman-Coulter, Mississauga, ON, Canada) automated liquid-handling instrument. Ribosomal RNA was removed with the Epicentre ScriptSeq Complete Gold Kit (Illumina) RNA-Seq libraries were prepared using the ScriptSeq Complete Epidemiology Next-Generation Sequencing (NGS) library kit (Illumina), barcoded, and combined into an RNA-Seq run on the Illumina MiSeq platform generating 201 bp paired-end reads (performed at the Pacific Biological Station, Nanaimo, BC, Canada).

A genomic assembly using the paired-end sequence reads from the partitioned library was assembled using the Supernova assembly algorithm (version 1.1.5, 10x Genomics, San Francisco, CA, USA). This produced an initial assembly with a scaffold N50 length of 16.79 Mbp (Table 1). To further improve this draft genome, we first assembled paired-end reads from the non-partitioned libraries from both of the individuals. Briefly, paired-end sequences from the same individual as the partitioned library were processed using Konnector (version 2.0) [3], which performs local de Bruijn graph assembly to generate pseudo-long reads representing complete DNA fragments. The resulting pseudo-long reads and remaining sequence reads that could not be incorporated by Konnector were assembled with ABySS (version 2.0.2, Canada’s Michael Smith Genome Sciences Centre) [4], using *kmer* values (*k*) between *k* = 60 and *k* = 120. Paired-end sequences from the second individual were incorporated in the later scaffolding stage of the ABySS assembly and did not contribute to the sequence content. The *k*100 ABySS assembly was determined to be the most contiguous based on the scaffold N50 length metric (N50 = 58,545, Table 1). We followed a scaffolding and gap-filling methodology similar to that of the recently published bullfrog genome [5]. Gaps in our initial, supernova draft assembly were filled with Cobbler (version 0.3, Canada’s Michael Smith Genome Sciences Centre) using parameters *-d* 100 *-i* 0.95 [6] utilizing contig sequences from the ABySS assemblies generated at three *kmer* values (*k*95, *k*100, *k*105). The subsequent gap-filled assembly was initially scaffolded with RAILS (version 1.2, Canada’s Michael Smith Genome Sciences Centre) using parameters *-d* 100 *-i* 0.95 [6] using scaffold sequences from the same three ABySS assemblies. Briefly, long sequences are aligned against a draft assembly using BWA-MEM (version 0.7.13), using parameters *-a -t* 16 [7], and the resulting alignments are parsed and inspected. When alignments satisfied our minimum alignment requirement (100 or more anchoring bases with over 95% sequence identity flanking each gap), we tracked the position and alignment orientation of each long sequence in the assembly draft. Sequence scaffolding was performed using the scaffolding algorithm from LINKS (version 1.8.5, Canada’s Michael Smith Genome Sciences Centre) [8], modified to automatically fill gaps with the sequence that informed the merge. The resulting assembly was re-scaffolded iteratively eight times with LINKS using parameters *-k* 26 *-l* 5 *-a* 0.3 *-d* 1,2.5,5,7.5,10,12.5,15,20 kbp, *-t* 10,5,5,4,4,3,3,2 -*o* 1 increment at each iteration, using scaffolds from all three ABySS assemblies. A final round of automated gap-filling with Cobbler using ABySS contigs was applied (same parameters as above). The genome scaffolding steps yielded an improved scaffold N50 length of 19.59 Mbp. Further improvement was achieved using Sealer (version 2.0.2, parameter *-k* at 60–200, step 10) [9], which used sequence contigs from the ABySS *k*100 assembly to close an additional 1059 sequence gaps. 

The final assembly (Table 1) comprises 2.327 Gbp of highly contiguous assembled genomic sequence with a scaffold N50 length of 19.59 Mbp. Analysis of the representation of highly conserved genes using Benchmarking Universal Single-Copy Othologs (BUSCO) [10] indicated that for 6253 genes, the complete and contiguous protein coding sequence was found in our assembly for 5669 genes (90.66%), whilst complete or fragmented sequences were found for 5915 genes (94.59%).

A transcriptome was obtained from de novo assembly of 75 bp paired-end chastity passed RNA-Seq reads sequenced on the Illumina NextSeq. Each library was assembled with ABySS (version 1.3.4, *k*38 to *k*74). The resulting assemblies within each library were merged using Trans-ABySS (version 1.4.10, Canada’s Michael Smith Genome Sciences Centre, Vancouver, BC, Canada) to produce a working transcript contig set (Table 2). MAKER (2.31.9, Yandell Lab, Salt Lake City, UT, USA) was used for the annotation and determination of protein coding potential of the genome assembly [11]. As part of its process, MAKER runs three ab initio gene prediction programs and uses experimental gene evidence to inform each. The gene predictions from each tool are then combined to produce a final annotated gene set. Within the MAKER framework, RepeatMasker [12] was used to mask low-complexity genomic sequences. The gene prediction programs AUGUSTUS [13], Snap [14], and GeneMark [15] were run within MAKER with the RNA-Seq assemblies and the annotated proteins of *O. orca* (Genbank accession ANOL00000000.2) were provided as experimental evidence. AUGUSTUS predictions were based on the included *Homo sapiens* training set of genes. Snap was trained using the CEGMA (version 2.5) predictive genes [16]. GeneMark was self-trained. These three sets of predictions were combined by MAKER to produce a final gene set of 29,581 genes, for which there was supporting experimental evidence. The RNA-Seq data provided evidence for multiple isoforms of some genes, and 38,561 transcripts were predicted from the 29,581 genes. The average predicted protein length was 402 amino acids. A full annotation of the genome is available from the Refseq website (https://www.ncbi.nlm.nih.gov/refseq/).

Overall, the contiguity of our assembly compares favourably with that of the *O. orca* (killer whale) genome, which reported a reconstructed genome size of 2.372 Gbp and scaffold N50 length of 12.735 Mbp [17]. With respect to assembly completeness, a similar number of BUSCO genes were present in a complete copy: 90.66% in the beluga and 91.46% in the orca. In addition, we estimated the degree of genome-wide sequence similarity between these two closely related organisms to be 97.87% ± 2.4 × 10^−7^% (mean ± standard deviation) using a *kmer*-based Bloom filter approach, as described by [18]. Our approach demonstrates the utility of microfluidic partitioned libraries to rapidly produce highly contiguous mammalian sized reference quality assemblies and we demonstrate how such assemblies can be further improved through the incorporation of assembled non-partitioned genomic libraries. We note that incorporating data from independent non-partitioned libraries provided only a modest overall improvement in the assembly, indicating that the partitioned libraries show no obvious bias. In the future, incorporating an independent assembly on the partitioned data might be an equally useful approach to improve assembly contiguity and eliminate the need for a second library. Our study also provides a deep transcriptomic resource that is profiled across multiple tissues.

## Figures and Tables

**Table 1:** Assembly statistics and gene content for the genome sequences reported in this study.

Assembly	Total Size (Gbp)	No. of Gaps	No. of Scaffolds	Scaffold N50 (bp)	Longest Scaffold (bp)	BUSCO Complete Genes	BUSCO Complete + Fragmented Genes
ABySS-pe	2.325 + 0.216% in gaps	210,782	102,940	58,545	997,316	4153 (66.42%)	4689 (74.99%)
Supernova	2.314 + 1.40% in gaps	30,858	8930	16.79 × 10^6^	78 × 10^6^	5667 (90.63%)	5911 (94.53%)
Rails/Cobbler	2.327 + 1.37% in gaps	26,898	6971	19.59 × 10^6^	95 × 10^6^	5669 (90.66%)	5915 (94.59%)
Sealer	2.327 + 1.36% in gaps	25,839	6971	19.59 × 10^6^	95 × 10^6^	5669 (90.66%)	5915 (94.59%)

BUSCO: Benchmarking Universal Single-Copy Orthologs.

**Table 2:** Transcriptome assembly statistics for all tissues studied and the read counts for each library.

Tissue	n	n:N50	Min	N80	N50	N20	Max	Sum	Read Count
Liver, dam	960,722	117,511	74	144	420	1542	47,312	246.3 × 10^6^	239.4 × 10^6^
Brain, dam	2,019,281	247,296	74	193	587	2013	18,656	691 × 10^6^	247.0 × 10^6^
Liver, daughter	854,394	99,263	74	145	555	1538	47,494	235.4 × 10^6^	241.2 × 10^6^
Brain daughter	2,258,624	260,327	74	198	653	2219	19,796	806.2 × 10^6^	270.4 × 10^6^
Lung, dam	1,170,674	374,225	162	201	282	504	5091	339.2 × 10^6^	25.6 × 10^6^
Mixed, dam	860,603	305,220	149	186	220	352	4751	208.2 × 10^6^	26.6 × 10^6^
Serum, dam	1,244,441	516,834	135	195	203	287	8034	281.5 × 10^6^	23.8 × 10^6^
